# Prevalence of active trachoma and associated risk factors among children in Gazegibela district of Wagehemra Zone, Amhara region, Ethiopia: community-based cross-sectional study

**DOI:** 10.1186/s40794-016-0022-0

**Published:** 2016-03-24

**Authors:** Zelalem Alamrew Anteneh, Wubante Yalew Getu

**Affiliations:** 1 Department of Epidemiology and Biostatistics, College of Medicine and Health Sciences, Bahir Dar University, Bahir Dar, Ethiopia; 2 Trachoma Control Program Coordinator in Organization for Rehabilitation and Development in Amhara (ORDA), Dessie, Ethiopia

**Keywords:** Trachoma, Risk factors, Children, Limited water

## Abstract

**Background:**

Trachoma continues to be hyperendemic in many rural areas of Ethiopia. The aim of this study was to determine the prevalence and associated risk factors of active trachoma among children in Gazegibela district, Ethiopia.

**Methods:**

A community-based cross-sectional study was conducted in April 2015 among children aged 1–9 years. Data were collected through an interview and eye examinations. Descriptive and logistic regression analyses were performed.

**Results:**

Among 601 children, 315 (52.4 %) were positive for active trachoma. Of these cases, 49.1 % were trachomatous inflammation-follicular and 3.3 % were trachomatous inflammation-intense. Children from households using rivers and ponds as their source of drinking water were more likely to develop active trachoma compared to those from households using water from springs or hand-dug wells (aOR = 2.9, 95 % CI: 1.70–4.81). Children from farming households were more likely to develop active trachoma (AOR = 3.3, 95 % CI: 1.02–10.65), as were children from housholds that lacked a latrine (aOR = 12.9, 95 % CI: 5.96–28.29). Children who washed their face only once a day were more likely to have active trachoma compared to those who washed for two and more times a day (aOR = 2.6, 95 % CI: 1.43–4.72).

**Conclusion:**

There is a high prevalence of trachoma among children from Gazegibela district. Trachoma remains a public health challenge in this region, requiring intervention from the government and other stakeholders.

## Background

Trachoma is a major public health challenge in developing countries. Caused by *Chlamydia trachomatis* and spread through contact with eye discharge from infected persons, it is the leading infectious cause of blindness in many regions [1]. Globally, trachoma affects 21.4 million people, of whom about 2.2 million are visually impaired and 1.2 million are blind. Despite changes brought about through socioeconomic development and disease control programmes, trachoma continues to be hyperendemic in many of the poorest rural areas of the world, especially in areas that have limited access to water and sanitation [2, 3].

According to 2011 WHO report, Ethiopia is one of the five countries of the world where 49 % of the global burden of active trachoma is concentrated [3]. According to the Ethiopian Demographic Health Survey 2007, the national prevalence of active trachoma, either trachomatous inflammation-follicular (TF) or trachomatous inflammation-intense (TI), for children 1–9 years was 40.1 %. The highest prevalence was registered in the Amhara region where 62.6 % of the cases were concentrated, followed by Oromia region with prevalence of 41.3 % [4]. In view of this health problem, the Ethiopia government signed the Declaration of Support for VISION 2020 initiative and developed its own 20 year strategic plan to eliminate trachoma in the country [5].

Various studies conducted in Ethiopia indicate that prevalence of trachoma varies significantly among children from region to region. The northern part of the country is disproportionately affected from the disease, with Wagehemera zone being one of the most severely affected [6–9]. Access to safe water, latrine utilization, and sources of fuel for cooking were found to affect the occurrence of active trachoma among children [9–11]. The aim of this study was to determine the prevalence and associated risk factors of active trachoma among children in Gazegibela district, Ethiopia.

## Methods

A community-based cross-sectional study was conducted among young children age 1–9 years in Gazegibela district. The district is one of the six districts in Wagehemera zone Amhara region.

### Sample size determination

The sample size of the study was determined using single population proportion formula, using an expected active trachoma prevalence of 24 % based on data from a previous study [8]. Giving any particular outcome to be within 5 % marginal error and 95 % confidence interval of certainty, based on these assumptions, the final sample size accounting for an additional 10 % for non-response was 616 children.

### Sampling strategy

Multistage sampling techniques were used to select participants from the six districts of Wagehemera zone, Gazegibela district using simple randomization. The district has nineteen administrative units (locally named Kebeles); four of the nineteen Kebeles were selected randomly. The sample size of 616 was distributed to each randomly selected Kebele proportional to the size of population. Thereafter, the sample size in each Kebele was divided by the number of households to determine the sampling fraction. Finally, systematic sampling method was used, and simple randomization was used among households that had more than one child aged 1–9 years.

### Measurement of the dependent variable

Trachoma was screened for clinically based on the WHO five sign grading system [12]. Eye examinations were performed by nurses trained in the diagnosis of eye diseases and care. Each eye was examined with the examiner sitting in front of study participant in the daylight using a binocular loupe (×2.5) and torch. Each eye was examined separately, with the examiner cleaning their hands with alcohol between each examination.

The presence or absence of signs of trachoma was made based on the following WHO grading criteria:

1. Trachomatous inflammation-follicular (TF), the presence of five or more follicles of >0.5 mm in the upper tarsal conjunctiva.
2. Trachomatous inflammation-intense (TI), Pronounced inflammatory thickening of the tarsal conjunctiva that obscures more than half of the normal deep tarsal vessels.
3. Trachomatous conjunctival scarring (TS), the presence of easily visible scaring in the tarsal conjunctiva.
4. Trachomatous trichiasis (TT), at least one eyelash rubs on the eyeball, evidence of recent removal of in turned lashes.
5. Corneal opacity (CO), easily visible corneal opacity over the pupil so dense that at least part of the pupil margin is blurred when viewed through the opacity.

Finally, the presence or absence of each sign of trachoma was recorded on data collection form for each study individual.

### Data collection procedures and data collection tools

Prior to the data collection, Wagehemera Zone Health Department and the Woreda Health Office were contacted for permission to conduct the interviews and eye examinations among children. Four data collectors performed the interviews, and four nurses (all integrated eye care workers (IECW)) performed the eye examinations. Training was given for both data collectors and integrated eye care workers.

The questionnaire was adopted from survey reports such as the Ethiopian Blindness and Trachoma Survey [13]. It was prepared in English and translated to local language, Amharic, and back translated to English to keep its validity. This system was pretested.

### Data processing and analysis

Prior to data entry, questionnaires were checked for errors and were coded. Data were entered into EPIinfo, and exported to SPSS for analysis. Univariate and bivariate analyses were computed to determine the frequency distribution and to test for the association between active trachoma and selected independent variables, respectively. Variables with *p*-value ≤0.20 in bivariate analysis were included in the multivariable logistic regression analysis. Confidence intervals (95 %) not containing one and a corresponding *p*-value <0.05 were considered statistically significant.

### Ethical approval

The study followed the guidelines of the Declaration of Helsinki. Ethical approval was obtained from the ethical review committee of GAMBY College of Medical Sciences, and letters of permission were obtained from Amhara Regional Health Bureau Technology Transfer Core Process and Gazegibela district health offices. Before the commencement of data collection, the objective of the study was clearly explained to each household head and verbal consent was obtained from them on behalf of the children. As most people living in rural Ethiopia are unable to read and write, the option for obtaining ethical consent orally was supported by ethical review boards and committees in Ethiopia. Treatment was prescribed for children diagnosed with active trachoma, and those with complicated cases and trichasis were referred to the nearby health centre for further care.

## Results

### Socio-demographic characteristics of study participants

Of the 601 children recruited in the study, 516 (85.9 %) were from a family headed by the males, and 407 (67.7 %) were from a family containing five or less members. Three hundred and fifty (58.2 %) households had greater than two children aged <10 years. Of the 601 children included in the study, 332 (55.2 %) were aged 1–4 years and the remaining 269 (44.8 %) were aged 5–9 years. See (Table 1).

**Table 1:** Socio-demographic characteristics of study participants in Gazegibela District, Wagehemera Zone, Northeast Ethiopia, April, 2015

Variables	Categories	Frequency	Percentages
Sex of HH head	Male	516	85.9
	Female	85	14.1
Marital status of HH head	Married	515	85.7
	Divorced	80	13.3
	Widowed	6	1.0
Religion of HH head	Orthodox Christian	596	99.2
	Muslim	5	0.8
Ethnicity of HH head	Amhara	518	86.2
	Agew	82	13.6
	Tigrie	1	0.2
Occupation of HH head	Farmer	544	90.5
	Marchent	57	9.5
Educational status of HH head	Unable to read and write	398	66.2
	Able to read and	168	28
	Grade 1 to 8	7	1.2
	Grade 9 to 12 and above	28	4.7
Family monthly income	<350 ETB	188	31.3
	351–450 ETB	155	25.8
	451–650 ETB	110	18.3
	>650 ETB	148	24.6
Family size	≥5	407	67.7
	> 5	194	32.3
Number of children <10 year in HH	1	251	41.8
	≥2	350	58.2
Number of rooms in living house	1	230	38.3
	≥2	371	61.7
Age of children	1–4 years	332	55.2
	5–9 years	269	44.8
Sex of child	Male	268	44.6
	Female	333	55.4

*HH* household

### Environmental factors

In this study, 480 (79.9 %) households cooked their food in a separate kitchen, whereas the other 121 (20.1 %) households cooked in the living area with an open fire. Rivers and ponds were used as water sources in 143 (23.8 %) of the households, and the majority of study participants (509 (84.7 %)) lived in families with average daily water consumption of 40–60 l/family/day. 478 (80 %) of households had latrines. See (Table 2).

**Table 2:** Environmental factors that might contribute to the occurrence of trachoma in Gazegibela District, Wagehemera Zone, Northeast Ethiopia, April, 2015

Variables	Categories	Frequencies	Percentages
Sources of water	Protected water source	458	76.2
	Unprotected water sources	143	23.8
Water consumption in liters/day	< 40 litters	36	6.0
	40–60 litters	509	84.7
	>60 litters	56	9.3
Cooking place	Kitchen	480	79.9
	In living room	121	20.1
Sources of energy for cooking	Fire wood	599	99.7
	Charcoal	2	0.3
Window in cooking room	Yes	385	64.1
	No	216	35.9
Methods of waste disposal	On disposal pit	321	53.4
	On farm	280	46.6
Presence of latrine	Yes	478	79.5
	No	123	20.5
Frequency of latrine use (*n* = 478)	Regularly	389	81.4
	Not regularly	89	18.6
Cattle ownership	Yes	466	77.5
	No	135	22.5
Shelter of cattle (*n* = 466)	Shelter constructed for cattle around the house	462	99.1
	In the same living room the family living	4	0.90

### Behavioral factors of the study participants

Some 443 (73.7 %) of the respondents reported that they washed their face ≥2 times per day, whereas the remaining 158 (26.3 %) washed their face only once daily. Only 274 (45.6 %) of respondents reported using soap to wash their face. See (Table 3).

**Table 3:** Behavioral factors of the study participants in Gazegibela District, Wagehemera Zone, Northeast Ethiopia, April, 2015

Variables	Categories	Frequency	Percentage
Frequency of face washing	Two and more times daily	443	73.7
	Once daily	158	26.3
Soap use during face washing	Yes	274	45.6
	No	327	54.4
Frequency of face washing using soap (*n* = 274)	Always	109	39.8
	Sometimes	165	60.2

### Prevalence of active trachoma among children age 1–9 years in Gazegibela district

The overall prevalence of active trachoma in children of age 1–9 years was 315 (52.4 %). Of these cases, 295 were TF and 20 were TI. The prevalence of active trachoma among children of age 1–4 and 5–9 years was 176 (55.9 %) and 139 (44.1 %) respectively. See Table 4.

**Table 4:** Prevalence of active trachoma among young children age1-9 years in Gazegibela district based on WHO grading system, April, 2015

Active trachoma	Frequency	Percentages (%)
TF	295	49.1
TI	20	3.3
Total	315	52.4

*TF* trachomatous inflammation (follicles), *TI* trachomatous inflammation (intense)

### Association between predictor variables and active trachoma among children in Gazegibela district

Bivariate logistic regression analysis was used to examine associations between socio-demographic, environmental, behavioral factors and active trachoma. Water sources, family monthly income, cooking place, presence of window in the kitchen, method of waste disposal, presence of latrine, latrine utilization, sex of child and frequency of face washing were associated with active trachoma (Table 5).

**Table 5:** The association between selected predictor variables and active trachoma among children in Gazegibela district, Wagehemera Zone, Northeast Ethiopia, 2015 (*n* = 601)

Variable	Trachoma(*n* = 601)			
	Yes	No	COR(95 % CI)	*P*-value
Education o head of HH				.001
Illiterate	228	170	1.00	
Read and write	77	91	0.6(0.44–0.91)	
Primary(1–8)	4	3	0.2(0.081–0.51)	
High school(9–12) and above	6	22	0.9(0.220–4.50)	
Water source				<0.001
Protected sources	220	238	1.00	
Unprotected sources	95	48	2.1(1.45–3.17)	
Monthly Income(Quartile)				<0.001
<=350 ETB	140	48	7.1(4.34–11.55)	
351–450 ETB	87	68	3.1(1.94–5.03)	
451–650 ETB	45	65	1.7(1.01–2.84)	
>650 ETB	43	105	1.00	
Occupation of head of HH				.104
Farmer	291	253	1.00	
Merchants	24	33	1.6(0.91–2.75)	
Family size				0.130
<=5	222	185	1.00	
>5	93	101	1.3(0.93–1.84)	
Where do the family cook				.011
In a kitchen	239	241	1.00	
Without kitchen	76	45	1.7(1.13–2.55)	
Presence of window in cooking room				<0.001
Yes	166	219	1.00	
No	149	67	3.4(0.56–21.12)	
Method of waste disposal				.001
Solid waste pit	148	173	1.00	
On farm	167	113	1.7(1.25–2.38)	
Presence of latrine				<0.001
Yes	203	275	1.00	
No	112	11	13.8(7.24–26.29)	
Ownership of cattle				.082
Yes	255	211	1.00	
No	60	75	2.5(0.26–24.21)	
Sex of selected child				<0.001
Male	76	192	6.4(4.49–9.18)	
Female	239	94	1.00	
Frequency of face washing				<0.001
Two or more times per day	196	247	1.00	
Once daily	119	39	3.8(2.56–5.78)	

*HH* household, *COR* crude odds ratio, *CI* confidence interval, *ETB* Ethiopian Birr

The effect of multicollinearity intra independent variables was tested using the standard errors of beta coefficients. The assumptions of logistic regression analysis were assessed using Hosmer and Lemeshow model fitness test, resulting *p*-value of 0.783. All variables with *p*-value of less than 0.2 in the bivariate result (Table 5) were entered in to multivariable logistic regression analysis using back ward stepwise methods of elimination. Water source, monthly family income, occupation, presence of latrine, sex of child, and frequency of face washing were significantly associated with the occurrence of active trachoma among children in the district (Table 6).

**Table 6:** Multivariable logistic regression analysis between predictor variables and active trachoma among children of age 1–9 years in Gazegibela district, Northeast Ethiopia, 2015

Variables	Active trachoma		OR(95 % CI for OR)	
	Yes (*n* = 315)	No (*n* = 286)	Crude	Adjusted
Water sources				
Protected sources	220	238	1.00	1.00
Unprotected sources	95	48	2.1(1.45–3.17)	2.9(1.70–4.81)*
Family monthly income				
≤350 ETB	140	48	7.1(4.34–11.55)	3.9(2.04–7.61)*
351–450 ETB	87	68	3.1(1.94–5.03)	1.9(1.06–3.68)*
451–650 ETB	45	65	1.7(1.01–2.84)	1.5(0.77–2.95)
>650 ETB	43	105	1.00	1.00
Occupation HH				
Farmers	291	253	1.6(0.91–2.75)	3.3(1.02–10.65)*
Merchants	24	33	1.00	1.00
Presence of latrine				
Yes	203	275	1.00	1.00
No	112	11	13.8(7.24–26.29)	12.9(5.96–28.29)*
Sex of child				
Male	76	192	1.00	1.00
Female	239	94	6.4(4.49–9.18)	6.1(3.85–9.61)*
Frequency of face washing				
Two or more times/day	196	247	1.00	1.00
Once/day	119	39	3.8(2.56–5.78)	2.6(1.43–4.72)*

*ETB* Ethiopian Birr, *HH* household, *AOR* adjusted odds ratio, *COR* crude odds ratio, Asterisk shows the variable is significant at *p*-value of 0.05 level in the multivariable logistic regression analysis

## Discussion

The aim of this study was to determine the current burden of active trachoma among children in Gazegibela district Ethiopia following the introduction of an integrated national prevention and control program for the disease. We found an overall prevalence of active trachoma among children aged 1–9 years of 52.4 %, the vast majority of which were TF.

Our findings are in agreement with a survey conducted in 2006 by Ethiopian Federal Ministry of Health (FMH) in ten zones of Amhara region, where the prevalence of active trachoma among children was 53.2 % [13]. However, the prevalence is slightly lower than was recorded in the Wagehemera zone in 2007 (60.1 %) [14], and in nearby South Sudan in 2005 (64.1 %) [15]. The lower prevalence in our study may be attributed to the activities provided by Ministry of Health and non-governmental organizations in the form of mass chemoprophylaxis and health education.

In contrast, the prevalence of trachoma in Gazegibela district is much higher than was found in similar studies from other parts of Ethiopia, such as in Baso Liben district, Gondar zuria district, Kersa district, Dangla district and Dera woreda where prevalences of active trachoma were 24.1, 23.8, 25.2, 12 and 15.6 % respectively [6, 8, 9, 16, 17]. This difference might be due to poorer infrastructure and low health service coverage in Gazegibela district, which is also an area that is repeatedly drought affected and one of the food insecure districts of the Amhara region. The prevalence of active trachoma in our study is also higher than reported from other African countries. For example, prevalences of active trachoma were 25.1 and 16.1 % in Malawi and Ghana respectively [18].

We found that the prevalence of active trachoma among children of aged 1–4 years was higher than among children aged 5–9 years. This might be due to greater exposure to dust particles in younger children, and poorer hygiene practices. This finding is in agreement with similar studies conducted in Kersa District, where children of lower age groups were more likely to be at risk of trachoma compared to children with high age groups [6].

The risk factors for active trachoma identified in our study have also been reported from other regions of Ethiopia and other African countries. This includes the use of unprotected water sources (rivers and ponds) [15], low monthly household income [6, 8], being from a farming household [8, 11], absence of a household latrine [8, 19], female gender [6, 15], and infrequent face washing [8, 9, 11, 20].

A major limitation of this study is the relatively small number of participants from only one of the six districts of the Waghemera zone. This is largely a result of our resource limitations. A larger sample size would have provided greater statistical power to enable more detailed investigation.

## Conclusions

The findings of this study revealed that about half of the total children screened for trachoma were positive for the disease. The finding implies that trachoma is still a major public health concern among children in the study area which demands further attention of the regional government and different stakeholders for intervention. The district health office should strength health extension packages and community lead total sanitation and hygiene (CLTSH) to reduce the burden of the disease. The health extension workers should also work in harmony with the community members to improve face washing habits of children. Besides, school based regular health education program needs to be implemented in the district in order to prevent the disease.

## Abbreviations

CLTSH: community led total sanitation and hygiene
IECW: integrated eye care worker
SAFE: surgery, antibiotic, facial cleanliness & environmental sanitation
TF: trachomatous inflammation, follicles
TS: trachomatous inflammation, intense
TT: trachomatous trichiasis

## References

[1] Water, Sanitation, & Environmentally-related Hygiene, Hygiene related disease. Center of disease prevention and control (CDC); 2009. Available from: http://www.cdc.gov/healthywater/hygiene/disease/trachoma.html. Accessed 9 Sept 2015.

[2] Trachoma. World Health Organization (WHO). Available from: http://www.afro.who.int/en/health-topics/topics/4421-trachoma.html. Retrieved 25 Mar 2015.

[3] Elimination of blinding trachoma: Only 10 years to go. Report of the Fourteenth Meeting of the WHO Alliance for the Global Elimination of Blinding Trachoma, Geneva, Switzerland, 19–21 April 2010.

[4] Yemane B, Alemayehu W, Abebe B (2007). Prevalence of active trachoma and trachomatous trichiasis at national and regional level. Ethiop J Health Dev.

[5] Keffyalew Gebremedhin. The Ethiopian observatory, Curing trachoma eyes & disease prevention in trachoma ‘endemic Ethiopia’: Ethics & fairness need to be guiding principles. Available from: http://ethiopiaobservatory.com/2015/02/20/curing-trachoma-eyes-disease-prevention-in-trachoma-endemic-ethiopia-ethics-fairness-need-to-be-guiding-principles/. Accessed 4 Sept 2015.

[6] Ejigu M, Kariuki MM, Ilako DR, Gelaw Y (2013). Rapid trachoma assessment in Kersa District, Southwest Ethiopia. Ethiop J Health Sci.

[7] The carter center. Prevalence and risk factors for malaria and trachoma in Ethiopia, Report of malaria and Trachoma Survey in Ethiopia. 2007.

[8] Ketema K, Tiruneh M, Woldeyohannes D, Muluye D (2012). Active trachoma and associated risk factors among children in Baso Liben District of East Gojjam, Ethiopia. BMC Public Health.

[9] Shiferaw D, Moges HG (2013). Risk factors for active trachoma among children aged 1-9 years in Maksegnit town, Gondar Zuria District, Northwest Ethiopia. Saudi J Health Sci.

[10] Shiferaw D, Moges HG. Risk factors for active trachoma among children aged 1-9 years in Maksegnit town, Gondar Zuria District, Northwest Ethiopia. Saudi J Health Sci. 2013;2:202-6.

[11] Harding-Esch mail EM, Edwards T, Mkocha H. Trachoma prevalence and associated risk factors in the Gambia and Tanzania, baseline results of a cluster randomized controlled trial. Published: Nov 02, 2010 doi:10.1371/journal.pntd.0000861. 10.1371/journal.pntd.0000861 PMC2970530 21072224

[12] WHO simplified trachoma grading system. Community Eye Health. 2004;17(52):68. PMC1705737 17491830

[13] Yemane Berhane, Alemayehu Worku, and Abebe Bejiga. National Survey on Blindness, Low Vision and Trachoma in Ethiopia by Federal Ministry of health, Addis Ababa, Ethiopia September 2006.

[14] The carter center. Prevalence and risk factors for malaria and trachoma in Ethiopia, a household cluster survey of trachoma prevalence and risk factors in Amhara region. 2007. Available at: https://www.cartercenter.org/resources/pdfs/news/health_publications/malaria/TCC_Malaria_Trachoma_HH_survey_Mar2008.pdf. Accessed 25 Oct 2015.

[15] Ngondi J, Onsarigo A, Adamu L (2005). The epidemiology of trachoma in Eastern Equatoria and Upper Nile States, southern Sudan. Bull World Health Organ.

[16] Gedefaw M, Shiferaw A, Alamrew Z (2013). Current state of active trachoma among elementary school students in the context of ambitious national growth plan: The case of Ethiopia. Health.

[17] Alemayehu M, Koye DN, Tariku A, Yimam K (2015). Prevalence of active trachoma and its associated factors among rural and urban children in Dera Woreda, Northwest Ethiopia: a comparative cross-sectional study. BioMed Res Int.

[18] World Health Organization (2001). Report of the six meeting of the WHO alliance for the global elimination of blinding trachoma.

[19] WHO. Trachoma Data Form World Health Organization Alliance for the Global Elimination of Blinding Trachoma, Twelfth Meeting Geneva, Switzerland: 28–30 April 2008; 1(8).

[20] Lemma E (2001). Prevalence and risk factors of trachoma among children of Wereilu Woreda, South Wollo administrative zone. Ethiop J Health Sci.

